# Complete Genome Sequence of Exfoliative Toxin-Producing Staphylococcus aureus Strain MSSA_SSSS_01, Obtained from a Case of Staphylococcal Scalded-Skin Syndrome

**DOI:** 10.1128/MRA.01335-20

**Published:** 2021-02-11

**Authors:** Eleonora Cella, Michael Z. David, Mohammad Jubair, Sarah L. Baines, David A. Pegues, Taj Azarian

**Affiliations:** a Burnett School of Biomedical Sciences, University of Central Florida, Orlando, Florida, USA; b Division of Infectious Diseases, University of Pennsylvania, Philadelphia, Pennsylvania, USA; c Department of Microbiology & Immunology, The University of Melbourne at The Peter Doherty Institute for Infection & Immunity, Melbourne, Victoria, Australia; d Department of Healthcare Epidemiology, Infection Prevention and Control, University of Pennsylvania, Philadelphia, Pennsylvania, USA; University of Rochester School of Medicine and Dentistry

## Abstract

Here, we announce the complete genome sequence of an exfoliative toxin-producing strain of Staphylococcus aureus sequence type 582 (ST582), isolated from a case of staphylococcal scalded-skin syndrome. The genome consists of a single circularized unitig with a total length of 2,792,190 bp carrying 2,699 genes. The genome is the basis for future epidemiological and genomic studies.

## ANNOUNCEMENT

Staphylococci that produce exfoliative toxins are the causative agents of staphylococcal scalded-skin syndrome (SSSS), or Ritter disease, a skin-blistering condition that affects children (1). Methicillin-sensitive Staphylococcus aureus strain MSSA_SSSS_01 was isolated according to CLSI guidelines and identified to the species level using matrix-assisted laser desorption ionization–time of flight mass spectrometry (MALDI-TOF MS) from a clinical case of SSSS in a hospitalized infant from Pennsylvania. A study waiver was obtained from the University of Pennsylvania Institutional Review Board.

For genome sequencing, the isolate was grown overnight (37°C, 200 rpm) in Bacto tryptic soy broth (BD, Heidelberg, Germany). Genomic DNA (gDNA) was extracted using the Qiagen DNeasy blood and tissue kit and phenol-chloroform extraction for high-molecular-weight gDNA using a protocol available online at dx.doi.org/10.17504/protocols.io.mrxc57n with the addition of 20 mg/ml of lysozyme to the lysis buffer. Size selection was performed using BluePippin with 0.75% PippinHT cassette. The quality and concentration of gDNA were assessed using the Agilent 4200 TapeStation system and Qubit 4 fluorometer.

Using an Oxford Nanopore Technology (ONT) MinION device, ligation (SQK-LSK109) and rapid (SQK-RBK004) sequencing kits, and an R9.4.1 flow cell, we produced 800 Mbp and 141 Mbp of data with mean read lengths of 12,138 bp (longest read, 75.1 kbp) and 2,852 bp (longest read, 123.2 kbp) and mean read quality of 11.2 and 10.5 for the ligation and rapid kit, respectively. We performed base calling using Guppy v0.5.1 with FAST mode and adapter trimming with Porechop v0.2. ONT reads were merged and filtered with Filtlong v0.2.0 using min_length 1000 and --target_bases 300000000. Data quality was assessed using NanoPlot 1.0.0 (2). Filtered ONT data had a mean read length of 15,807 (*N*_50_, 16,493 bp) and mean read quality of 12.2. We then used the Illumina MiSeq platform with a Nextera Flex kit and V3 flow cell to produce 430 Mbp (862,519) of 2 × 250-bp paired-end reads (>153× idealized coverage). Illumina data were filtered using Trimmomatic v0.39 with SLIDINGWINDOW:10:20 MINLEN:31 TRAILING:20 (3). A long-read assembly was generated from Nanopore data using Unicycler v0.4.8 with default settings, which resolved a single circularized unitig (4). For refinement, Snippy v4.6.0 (https://github.com/tseemann/snippy) with bcftools v1.10.2 was used to map the Illumina data to the draft long-read assembly. The final assembly has a total length of 2,792,190 bp and a G+C content of 32%. The NCBI Prokaryotic Genome Annotation Pipeline was used to annotate the genome, identifying 2,699 protein-coding sequences, 59 tRNAs, 7 5S rRNAs, 6 16S rRNAs, and 6 23S rRNAs (5).

Multilocus sequence typing (MLST) and KmerFinder tools (https://cge.cbs.dtu.dk/) were used to identify MLST and the closest matching reference genome (6–8). Characterization of prophages was performed using PHASTER (9). MSSA_SSSS_01 was identified as sequence type 582 (ST582) belonging to clonal complex 15 (CC 15) and most closely related to strain 08-02119 (GenBank accession no. NZ_CP015645). The strain is significant for the presence of a 42.3-kb ϕETA phage (830,002 to 873,202 bp; G+C content, 34.95%) closely matching *Staphylococcus* phage B166 (NC_028859.1) and harboring the exfoliative toxin A (*eta*) gene (10). Expression of *eta* was confirmed using quantitative reverse transcriptase PCR (RT-PCR) as previously described (11). In addition, MSSA_SSSS_01 also possesses a 45.6-kb Panton-Valentine leukocidin (ϕPVL) phage (1,541,971 to 1,587,596 bp; G+C content, 33.02%) most closely related to *Staphylococcus* phage phi2958PVL (NC_011344.1) (10).

### Data availability.

This whole-genome shotgun project has been deposited at DDBJ/ENA/GenBank under the accession no. PRJNA662727. The BioSample accession described in this paper is SAMN16093280. The SRA accessions are available at SRX9565383, SRX9565384, and SRX9565385.
